# Automated, Consistent, and Even-Handed Selection of Active Orbital Spaces for Quantum Embedding

**DOI:** 10.1021/acs.jctc.3c00653

**Published:** 2023-09-29

**Authors:** Elena Kolodzeiski, Christopher J. Stein

**Affiliations:** Technical University of Munich, TUM School of Natural Sciences, Department of Chemistry, Lichtenbergstr. 4, Garching D-85748, Germany

## Abstract

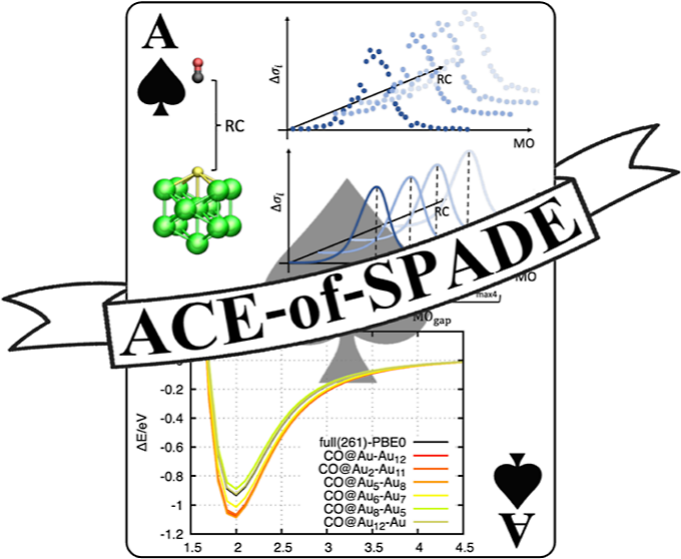

A widely used strategy to reduce the computational cost of quantum-chemical calculations is to partition the system into an active subsystem, which is the focus of the computational efforts, and an environment that is treated at a lower computational level. The system partitioning is mostly based on localized molecular orbitals. When reaction paths or energy differences are to be calculated, it is crucial to keep the orbital space consistent for all structures. Inconsistencies in orbital space can lead to unpredictable errors on the potential energy surface. While successful strategies to ensure this consistency have been established for organic and even metal–organic systems, these methods often fail for metal clusters or nanoparticles with a high density of near-degenerate and delocalized molecular orbitals. However, such systems are highly relevant for catalysis. Accurate yet feasible quantum-mechanical ab initio calculations are therefore highly desired. In this work, we present an approach based on the subsystem projected atomic orbital decomposition algorithm that allows us to ensure automated and consistent partitioning even for systems with delocalized and near-degenerate molecular orbitals and demonstrate the validity of this method for the binding energies of small molecules on transition-metal clusters.

## 1 Introduction

In recent years, as worries about climate change and environmental pollution have grown, heterogeneous catalysis has gained increasing attention since these catalytic transformations are among the most energy-consuming industrial processes.^1,2^ Improvements in these catalysts’ selectivity and efficiency are thus extremely desirable not only from a commercial point of view but also for addressing environmental challenges such as the reduction of greenhouse gas emissions.^3–6^ Metal nanoparticles are excellent candidates as catalysts since they provide a large surface area with a high number of possible active sites, enabling efficient and effective catalytic reactions.^7–10^ Since their catalytic abilities rely on various intrinsic and environmental conditions, it is essential to derive a detailed understanding of their electronic properties and operating mechanisms.^11–15^

In this regard, computational modeling has become a key component. The accurate description of complex electronic structures is a significant challenge, even for modern computational methods.^12,14,16^ Although wave function theories such as configuration interaction,^17,18^ coupled cluster,^19,20^ or perturbative post-Hartree–Fock^21,22^ methods allow achieving highly accurate and systematically improvable results, they are numerically too expensive for large systems.^23,24^ In contrast, Kohn–Sham Density Functional Theory^25^ (KS-DFT) provides a computationally more efficient approach at the price of substantial limitations due to the approximation of the exchange functional.^26^ The choice of the exchange functional has a significant impact on accuracy and varies depending on the system. In particular, the description of chemical reactions on transition-metal complexes or nanoclusters, which exhibit (near-)degenerate electronic character, is known to be error-prone.^27^

In the past decades, embedding methods have gained increasing attention.^28,29^ These methods are designed to precisely describe chemical reactions at a low computational cost. This is achieved by separating the system into an active subsystem, which is the focus of computational efforts, and an environment that is treated with a more approximate method. Versatile quantum embedding schemes such as QM/MM,^30^ implicit solvents,^31,32^ ONIOM models,^33,34^ density matrix embedding theory,^35^ Green’s function embedding,^36–38^ frozen density embedding (FDE), and subsystem DFT^39–46^ among many others^47–49^ have been developed.

Projection-based embedding theory (PBET) is a subsystem DFT approach which offers a robust, accurate, and simple framework for DFT-in-DFT and even WF-in-DFT embedding.^46,50^ In PBET, the subsystem partitioning is based on conventional orbital localization procedures such as Pipek–Mezey^51^ (PM) or intrinsic bond orbitals^52^ (IBOs), assigning molecular orbitals (MOs) to atoms, which correspond to the active or environmental subsystem, respectively. The partitioning of the orbital space based on such localization schemes has been termed manual selection (MS).^53^ However, during chemical reactions, the nature of the selected MOs might change and, with it, the partitioning of the active and environmental subsystems. This might lead to unphysical discontinuities in the potential energy surface (PES).^54–56^ To overcome this issue, so-called “even-handed” selection schemes have been proposed.^53,57,58^ The common idea of these methods is to identify MOs in the active region which are relevant for describing the chemical reaction and to form a consensus set of these MOs, which are then used for all embedding calculations along the reaction pathway. This eliminates discontinuities in the PES and provides an accurate evaluation method for energies. Automated approaches, such as the direct orbital selection (DOS) scheme invented by Bensberg and Neugebauer,^53,58^ have been developed for partitioning the molecular system most efficiently and accurately. We note, however, that these orbital selection approaches are all based on conventional localization schemes, which tend to strongly localize the MOs such that contributions from only one or two atoms are dominant.^55^

As an alternative, another elegant active orbital space selection scheme has recently been published by Claudino and Mayhall, which has been termed subsystem projected atomic orbital decomposition (SPADE).^55^ In this approach, again, a set of atoms is manually selected and allocated to the active subsystem. Based on this selection, the MOs are projected onto orthogonalized atomic orbitals of the active subsystem. Subsequently, singular value decomposition (SVD) is applied to the subsystem coefficient matrix of the active system. The transformation matrices derived from the SVD allow the transformation of the canonical MOs to a set of localized SPADE orbitals. The largest gap between consecutive singular values defines the most suitable system partitioning. This approach is, in contrast to conventional localization schemes, independent of any externally set parameters or thresholds. In contrast to conventional orbital localization procedures (such as IBO or PM), which produce MOs with almost atomic-orbital character, the SPADE algorithm localizes more broadly, obeying the subsystem boundaries.^55^ Further, studies have shown that this approach is more robust to the variation of a reaction coordinate than conventional localization schemes.^55^ However, despite the increased robustness, discontinuities cannot generally be excluded.^59^

Apart from these active-space selection schemes for quantum embedding, a variety of automated and semiautomated active orbital space selection schemes have emerged in the context of multiconfigurational calculations.^60–66^ Despite the common goal of suitable active-space selection, these methods show substantial differences with regard to their application areas. In multireference calculations, the primary objective is to capture all essential static correlations in the active orbital space. In contrast, in quantum embedding, we need to include all electronic contributions in the active subsystem for which we expect a significant change due to either a chemical reaction or an external stimulus. The partitioning is therefore close to an atomic partitioning, whereas selection schemes for multiconfigurational electronic-structure methods usually focus on the valence orbitals. However, it should be noted that the SPADE algorithm is very similar to the atomic valence active space^67^ scheme in that the atomic orbital overlap in conjunction with a singular value decomposition is used to define the active space for multiconfigurational calculations.

Although significant achievements have been made in the development and application of different selection schemes in PBET over the past decade, most of the reported studies focus either on organic systems or on small transition-metal complexes.^50,57,68–73^ Systematic studies dealing with energetically (near-)degenerate many-electron systems such as metal nanoclusters are so far limited. In particular, the application of the traditional localization schemes to metal nanoclusters is questionable since the MOs of such systems are strongly delocalized. Although there is some evidence that the regionally localizing SPADE algorithm might be most suitable for partitioning nonlocalized MOs, a detailed analysis is missing. Therefore, a comprehensive picture of the reliability of different active-space selection schemes on metal nanoclusters is required in order to expand the powerful PBET method to this class of systems.

Here, we report a systematic study analyzing the suitability of different active orbital selection approaches for the calculation of the PES of small molecules interacting with metal nanoclusters. We show that due to the delocalized nature of the MOs, orbital selection schemes, which are based on traditional localization schemes, result in enormous energetic discontinuities along the reaction pathway. The SPADE algorithm, by contrast, is more robust but still suffers from discontinuities in challenging cases. However, we show that this issue can be solved by utilizing an MO selection based on the singular value hierarchy. Our study shows that the SPADE algorithm consistently prioritizes the most relevant MOs even for (near-)degenerate orbitals, which allows us to trace the evolution of MOs along the reaction pathway. We exploit this finding to select a consistent set of MOs throughout the entire trajectory, converting the SPADE algorithm into an evenhanded approach that successfully eliminates all discontinuities. Although this approach solves the discontinuity problem, the question of its accuracy is still open. Our study demonstrates that simply increasing the number of actively treated molecular orbitals in the active system does not necessarily lead to a more accurate description of the dissociation curve. This behavior prevents systematic improvements and error estimations, which are highly required to increase the predictive power of these QM/QM embedding methods. To overcome this issue, we developed an automated and consistent even-handed partitioning approach for systems with (near-)degenerate MOs based on the SPADE algorithm and termed **ACE-of-SPADE**. We demonstrate the validity of this method for small molecule binding energies on transition metal clusters. The ACE-of-SPADE approach adequately balances the size of the active system and its accuracy.

Our study effectively addresses the challenges regarding system partitioning that come with the application of the PBET method to metal nanoclusters. The developed ACE-of-SPADE algorithm allows for an automated and efficient selection of active orbitals, which leads to reliable and systematically improvable results. This study shows how PBET can successfully be applied to strongly correlated and (near-)degenerate systems and makes therefore a significant contribution to the computational research of heterogeneous catalysis.

## 2 Computational Details

For this study, an Au_13_ nanocluster with a bonded carbon monoxide molecule (CO@Au_13_) has been studied as a prototypical test system. Before running the embedding calculations, a geometry optimization has been conducted using Q-Chem 5.4^74^ with the PBE0 functional^75^ and the def2-SVP basis set.^76^ The embedding calculations are performed with an adapted version of the quantum chemistry package Serenity.^77^ All embedding calculations are conducted as a Huzinaga-type top-down PBET in FDE approximation,^78^ using, unless otherwise stated, PBE0 for the active subsystem and local-density approximations (LDA)^25,79^ for the environment. The level shift parameter is set to 1.0 E_*h*_. For the embedding calculations, the def2-SVP basis set is applied as well. For the manual orbital selection schemes, IBO localization is used. The Mulliken net population threshold is set to τ_Mulliken_ = 0.4 to separate active from environmental MOs. When running the DOS scheme, the automated orbital alignment strategy of Bensberg and Neugebauer is applied.^53^ For a comparison of the MOs, the selection thresholds τ_kin_ and τ_loc_ must be defined. In this work, they are set equal to (τ_DOS_ = τ_loc_ = τ_kin_). To modulate the size of the active space, these parameters are varied in a range between 0.05 and 1. The algorithm used to calculate partial charges is based on the shellwise accumulation of intrinsic atomic orbitals.^52,53^

The SPADE algorithm is used as implemented in the Serenity program version 1.4. The initial orbitals considered in the selection scheme are the Kohn–Sham orbitals obtained from an SCF calculation for the entire supersystem with the same functional that is used for the calculation of the environment.

The structures along the reaction pathway are created based on the PBE0-optimized CO@Au_13_ complex. The vector pointing from the closest Au atom to the C atom defines the direction in which the CO substrate is iteratively moved without modification of the CO bond length (Figure S6). Starting from a distance of 1 Å between the substrate and metal nanocluster, the substrate is moved along the predefined vector for 40 steps with a step size of 0.1 Å.

Scripts and modifications to Serenity 1.4 required to run our proposed active-space selection algorithm are provided in the Supporting Information.

## 3 Results and Discussion

### 3.1 Assessing the Quality of Active Space Selection Schemes for Metal Nanoclusters

**Figure 1:**
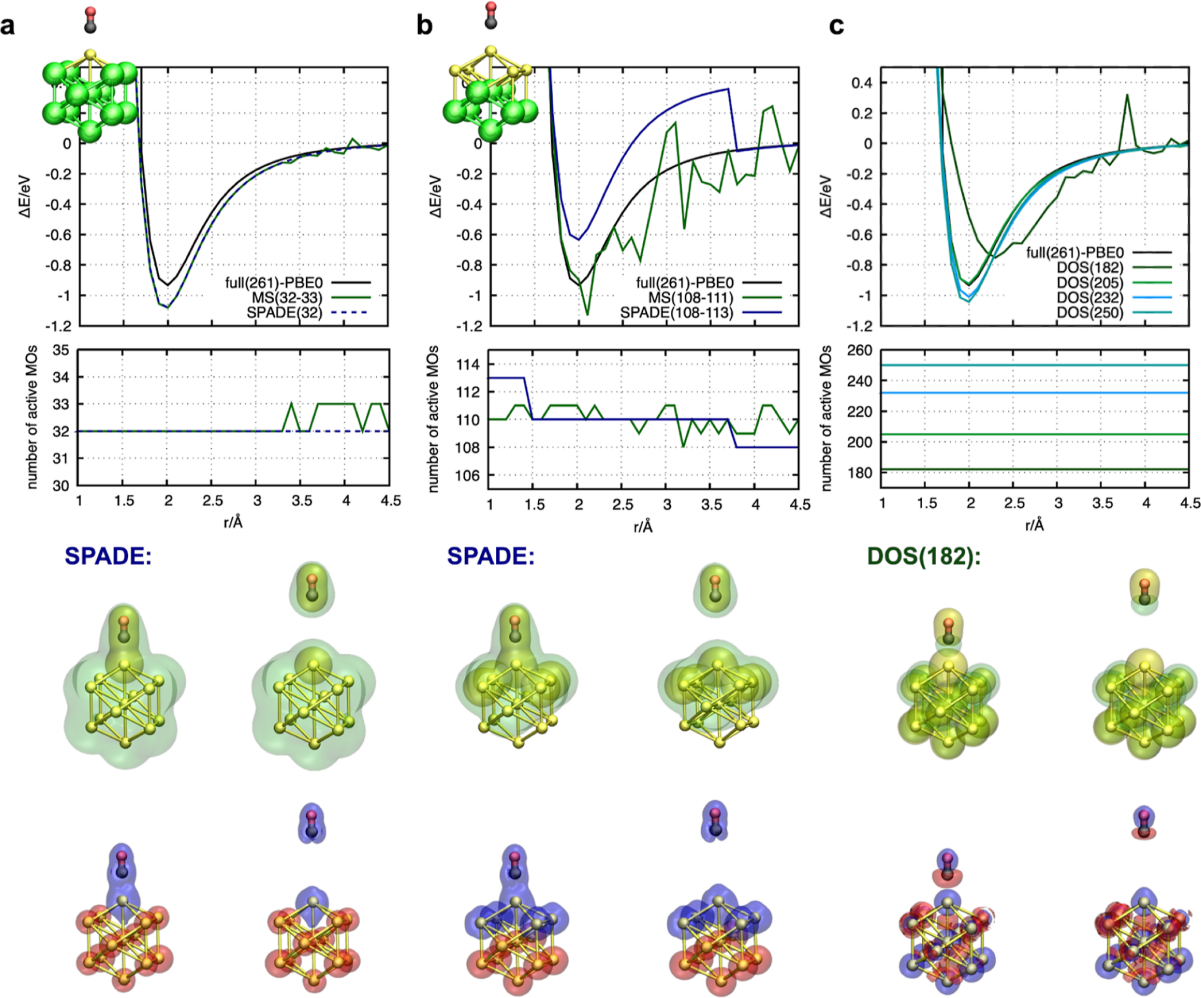
Impact of different active-space selection schemes on the dissociation curve of CO@Au_13_. (a,b) Dissociation curve calculated by embedding a CO@Au/CO@Au_5_ cluster as the active subsystem into the Au_12_/Au_8_ environment, respectively. (c) Dissociation curve using the DOS approach, considering different sizes of actively treated molecular orbitals. The density plots below show the density of the active subsystem (yellow) and the environmental subsystem (green) at the isovalue of 0.025 au for two different configurations along the reaction pathway. The left image in each subfigure corresponds to the configuration at *r* = 2.0 Å and the right image at *r* = 3.9 Å. In the bottom plots, the charge differences are depicted, comparing the density distribution of the pure PBE0 calculation with the density distribution after embedding: (ρ_diff_ = ρ_pure-PBE0_ – ρ_PBE0-in-LDA_). The blue areas highlight positive charge accumulation, while the red areas highlight negative charge depletion zones. The isovalues are 0.1 in (a,b) and 0.001 in (c).

**Figure 2:**
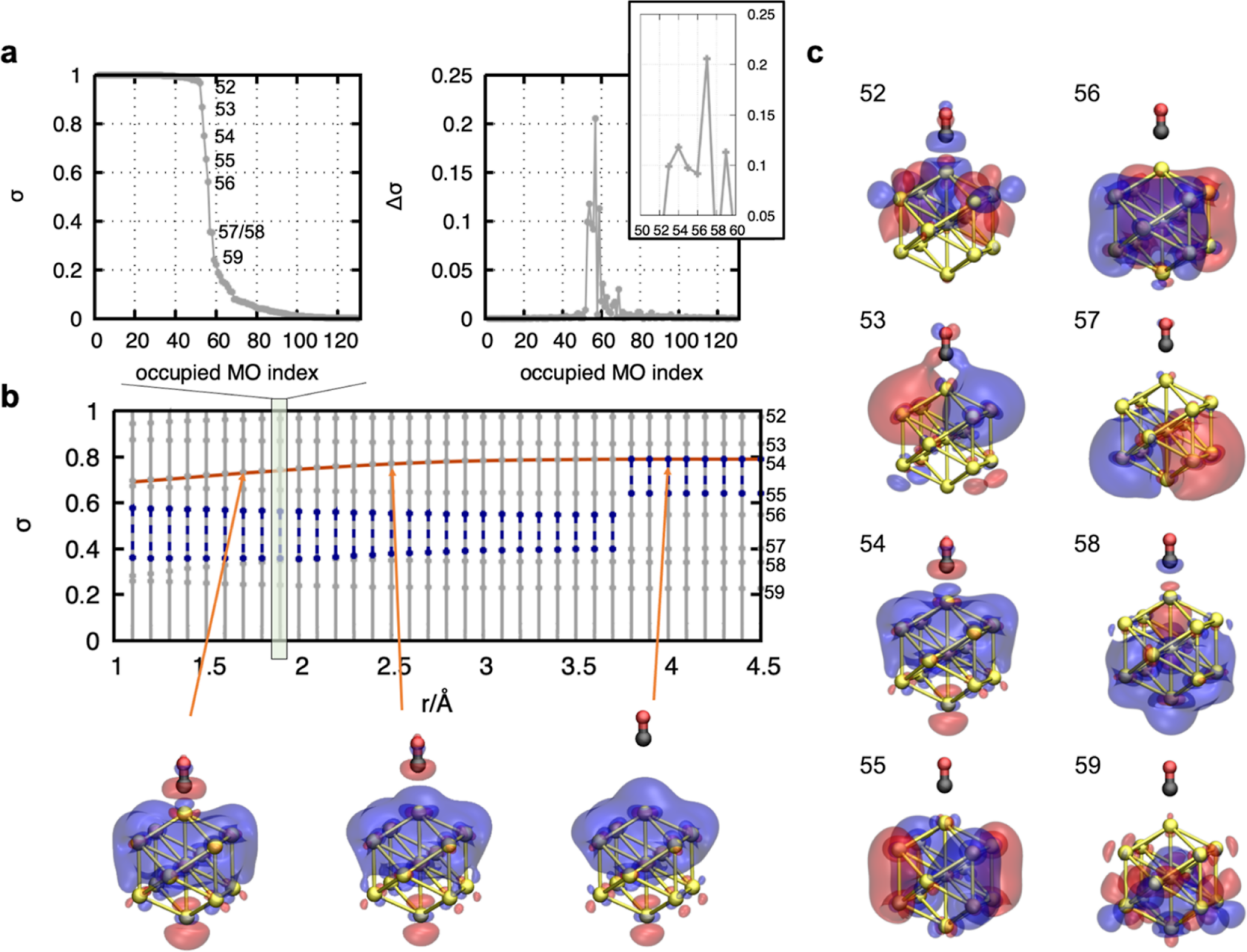
(a) Plots of the singular value σ_*i*_ distribution and the difference of consecutive MOs with respect to the MO index (Δσ_*i*_ = σ_*i*_ – σ_*i*–1_) for the CO@Au_5_-in-Au_8_ nanocluster. The inset in the right plot shows a zoom-in into the multipeak structure of the Δσ_*i*_ curve. (b) Evolution of the singular values along the reaction pathway. At each point *r* of the reaction coordinate, the singular values of MO with index 52–59 are shown by the gray dots. The gray lines connecting the singular values are printed as a guide to the eye, highlighting the affiliation of the singular values to the respective reaction coordinate. The blue-colored line fragments mark the largest gap between adjacent MOs, used in automated SPADE for system partitioning. The evolution of the MO with index 54 (highlighted by the orange curve) is shown by the three exemplary plots below. The Au atoms are colored yellow, the C atom in black, and the O atom in red. (The alpha MOs are shown by the blue) and the beta MOs by the red isosurfaces with isovalues of 0.015 and −0.015, respectively. (c) Illustration of the MOs with index 52 to 59 at *r* = 2.0 Å. The same color coding as in (b) is used.

First, we evaluate the quality of different active-space selection schemes for application on metal nanoclusters. Therefore, we compare the dissociation curves of CO@Au_13_ calculated using PBET with different active-space selection schemes with the dissociation curve obtained from a pure PBE0 calculation (see Figure 1). An intuitive choice for the system partitioning is to assign the substrate and the closest Au atom to the active subsystem, while all of the other Au atoms constitute the environment. The MOs of the active subsystem can then be obtained either from the MS scheme or from the SPADE algorithm. Figure 1a shows the dissociation curves which result from such a partition. The MS scheme leads to discontinuities at larger Au–C distances (*r* > 3.3 Å) due to fluctuations of the active orbital space size between 32 and 33 active MOs. In contrast, the SPADE algorithm is more robust regarding fluctuations of the actively treated MOs, leading to a smooth and continuous energy curve along the entire reaction pathway. However, the binding energy is overestimated by around 0.15 eV with respect to the pure PBE0 reference calculation for both selection schemes. In the next panel (Figure 1b), the number of actively treated atoms is manually increased, and the next layer of the metal cluster is included in a CO@Au_5_ active subsystem. For this system partition, both the MS and the SPADE algorithms show discontinuities. The MS approach fluctuates between 108 and 111 actively treated orbitals, leading to pronounced energy fluctuations along the reaction pathway. The origin of these strong fluctuations is the large number of delocalized MOs in the metal nanocluster. Figure S7 shows exemplary MOs after the application of the IBO localization procedure. Despite the application of traditional localization schemes, the MOs are still delocalized over the entire system. This, combined with the large number of (near-)degenerate orbitals, leads to multiple equivalent localized MOs. Even for slight structural changes, localization might lead to different sets of localized MOs. The unique identification of a consistent set of MOs along the reaction pathway is therefore not possible. The dissociation curve calculated with the SPADE algorithm is more robust. Nevertheless, a large energetic shift occurs at 3.7 Å. At this point, the number of active MOs drops from 110 to 108. Note, this effect is not related to the specific molecular structure or method used for the initial orbital calculation of the entire system but rather a general phenomenon that can occur for systems with delocalized MOs (see Figures S8 and S9). Figure 2b illustrates the evolution of the singular value distributions along the reaction pathway. In line with the discontinuities in the energy curve, the largest gap between adjacent MOs—which determines the MO selection—changes at 3.7 Å and causes a change in the active-space size. Although the position of the largest gap changes, it should be highlighted that this approach allows tracking the evolution of the MOs. The SPADE algorithm thus provides a consistent identification framework, which is required for the development of an even-handed scheme for many-body systems with many (near-)degenerate orbitals.

This aspect is discussed in more detail in Section 3.2. Apart from this, Figure 1c illustrates the performance of the DOS approach used to enforce an even-handed localization protocol based on conventional localization methods. In contrast to the previously discussed active-space selection approaches, a predetermination of active atoms is not required here. In this approach, active MOs are selected automatically based on the changes in their composition over the reaction trajectory. After the identification of relevant orbitals, this consensus set is used for all of the embedding calculations. Although such an even-handed approach should promise a smooth and continuous energy potential landscape, significant problems arise when applied to the metal nanocluster. Using recommended thresholds (τ_DOS_ = 0.05)^53^ for the comparison algorithm leads to active spaces in which almost all MOs are considered. This effect arises from the previously discussed observation that the localization schemes might converge to different sets of MOs for slightly modified structures. These inconsistencies are accidentally associated with MO changes along the reaction coordinate. Consequently, all of these MOs are considered active. The resulting curves are smooth, but the advantage of increased computational efficiency due to small active MO spaces is lost. In this regard, increasing the threshold allows for a reduction in the size of the active system. Therefore, unconventional parameters up to τ_DOS_ = 1 are applied. This reduces the active orbital size to 182 MOs, which is still more than half of all MOs. Further, with such large thresholds, the even-handedness of the selection procedure fails, which is manifested in several discontinuities along the reaction curve. This has been observed previously for large thresholds in the DOS procedure.^53^

The analysis has shown that active-space selection schemes based on localization algorithms such as IBO, which produce strongly localized orbitals, introduce large errors when analyzing the molecular properties of metal nanoclusters along a reaction pathway. The SPADE algorithm is less error-prone since it localizes more broadly, respecting the selected active subsystem and therefore keeping the delocalized character of the molecular orbitals to some degree. These properties can also be observed by analyzing the charge distributions of the active and environmental systems. Figure 1a–c (bottom panel) shows the density distribution of the active subsystem (yellow) and the environmental subsystem (green), obtained from the SPADE approach (a,b) and the DOS approach (c). Interestingly, the density distributions of the molecular environment obtained from the SPADE approach cover the entire molecular system. The molecular density of the environment around the active region is caused by the delocalized nature of the molecular orbitals. Although the orbitals that contribute most to the active system are identified and considered active, the accumulation of the minor contributions of the remaining delocalized orbitals assigned to the environment results in this density distribution. However, as shown in the charge difference plot (bottom row in Figure 1), which compares the total density of both subsystems with the target density obtained from the full PBE0 reference calculation, the accumulated density of both systems around the active region is overestimated, while the electron density in the environment is depleted.

### 3.2 Active-Space Convergence

**Figure 3:**
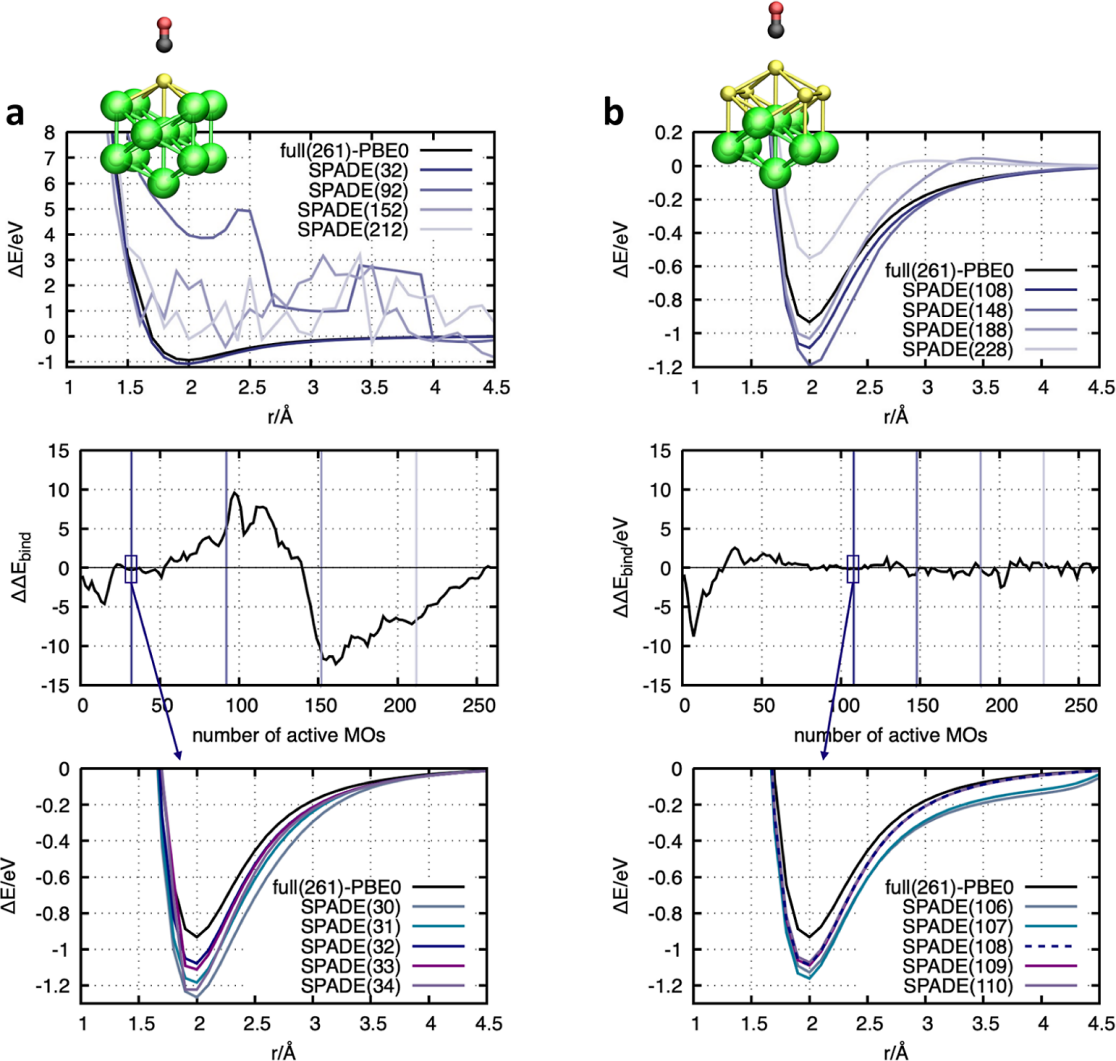
Impact of the active-space selection on the binding energy Δ*E* between an Au_13_ nanocluster and a CO molecule. The top image shows the binding energy Δ*E* with respect to the reaction coordinate *r*, which measures the distance between the C atom and the closest Au atom of the nanocluster. The insets show the structure of the CO@Au_13_ nanocluster. The green-colored Au atoms represent the molecular environment. The yellow Au atoms and the black C and red O atoms are treated as active. Orbitals of the environmental system are optimized on the LDA level, and orbitals of the active system are optimized on the PBE0 level. The panel in the middle shows the error in the binding energy (ΔΔ*E*_bind_ = Δ*E*^min^ – Δ*E*^0^) calculated based on the energies of configuration at *r* = 2.0 Å (Δ*E*^min^) and *r* = 3.9 Å (Δ*E*^0^). Only configurations with a multiplicity of 1 or 2 are considered. The vertical lines highlight the active space used for calculating the binding energy curve above. The bottom panels show dissociation curves calculated on the basis of MOs for a small window around the original SPADE partition.

The SPADE methodology enables a largely robust and accurate selection of the active MO space. Further, the SPADE algorithm retains some degree of delocalization for the orbitals of the active subsystem and introduces a ranking based on singular values. This allows the evolution of MOs along the reaction pathway to be analyzed and provides a possibility for uniquely identifying the relevant MOs over the course of a trajectory. This can be exploited to derive an even-handed approach based on the SPADE algorithm.^59^ However, embedding calculations promise to balance high accuracy and computational efficiency. Therefore, it is crucial to investigate the convergence behavior between the selected active-space size and the resulting errors. To study this, the dissociation curve is calculated by applying the SPADE algorithm to two different active clusters (CO@Au-in-Au_12_ and CO@Au_5_-in-Au_8_). For both cluster configurations, different sizes of the active MO sets are considered. Along each curve, the number of MOs is kept fixed (see Figure 3, top panels). The active orbitals are selected with respect to their index and ranked according to their singular values. Contrary to our expectations, increasing the active-space size does not necessarily increase the accuracy of the embedding calculation. In the case of the CO@Au-in-Au_12_ embedding calculation, discontinuities occur for large active spaces, indicating that the selection approach is no longer even-handed. The size of the hierarchically ordered MOs depends on the number of active atoms. In the case of CO@Au-in-Au_12_, only 118 MOs are considered during the singular value decomposition. Selecting MOs beyond this limit leads to discontinuities since they are not included in the localization scheme. Further, even calculations based on active-space sizes lower than 118 might be error-prone. Although MOs are prioritized by the singular value decomposition, many MOs have singular values close to 0 (see Figure S10). For such low numbers, the ordering is inconsistent between different structures, which results in fluctuations of the energies along the dissociation curve (see the energy curve based on 92 active MOs in Figure 3a). The best result for the CO@Au-in-Au_12_ configuration is achieved by selecting the active space with respect to the largest gap between consecutive MOs, leading to a constant active orbital space with 32 active MOs. The situation for the CO@Au_5_-in-Au_8_ (Figure 3b) configuration is similar. Increasing the number of active MOs may reduce accuracy. The best result is obtained for 108 active MOs, which corresponds to the average number of active MOs selected by SPADE along the trajectory.

This study shows that active-space sizes can be categorized into three regimes: In the first regime, active-space selection is attempted for orbitals with 0 < σ_*i*_ < 1. This is the region where the automated SPADE algorithm partitions the systems. Keeping the number of MOs in this regime constant leads to an even-handed orbital selection scheme. In the second regime, active space partitioning is applied to MOs that have similar singular values very close to 0. Since small (numerical) fluctuations can change the MO ordering, it cannot be ensured that a consensus set of the same MOs is used along the trajectory. The last regime for active MO sizes is an attempt to select orbitals that were not subjected to the singular value decomposition. The MOs in this regime are not localized. Consequently, the uniformity of MOs according to the singular value hierarchy cannot be established.

To get further insights into the dependence between the accuracy of the binding energy and active-space size, the errors of the binding energy with respect to the full PBE0 calculation have been calculated (see Figure 3, central plot). The plots for both systems, CO@Au-in-Au_12_ and CO@Au_5_-in-Au_8_, show nonmonotonic and nonconverging behavior. The lowest errors are obtained from a small range corresponding to the first regime (see the highlighted rectangles in Figure 3, central plot). For CO@Au-in-Au_12_, the best result in this transition area (0 < σ_*i*_ < 1) is achieved by following the largest gap selection of the original SPADE (see Figure 3a, bottom). Note that even small deviations in the active-space size (±1 active MO) affect the accuracy significantly (see Figure 3a, bottom). For the CO@Au_5_-in-Au_8_, a selection based on the largest gap is not straightforward since the gap position changes along the trajectory. Due to the active atom selection partitioning of the covalently bound metal nanocluster, many MOs have singular values in the transition region. Here, the best partitioning is obtained by the average position of the largest gaps along the trajectory. In this way, all the most favorable partitions of all structures are taken into account (see the dissociation curve in the bottom panel of Figure 3b calculated based on 108 active MOs). Analogous to the CO@Au-in-Au_12_ configuration, small fluctuations in the size of active orbital spaces (±1 active MO) lead to an adaption of the energy profile (see Figure 3b bottom). However, the deviations in the errors are smaller compared to those of CO@Au-in-Au_12_, due to the larger active system defined by the predefined active atoms. The individual MOs contribute less to the CO interaction of the active system due to the extended cluster size. Additionally, it should be mentioned that with an increasing number of active atoms, the errors of the different energy contribution terms decrease. This observation makes the approach more robust toward errors due to slight changes in the number of actively treated MOs. This will be discussed in more detail in Section 3.4.

This analysis shows that despite the prioritization of MOs according to the SPADE algorithm, no convergence with respect to the active system size is achieved. In contrast, we could show that the most reliable results concerning consistency and accuracy can be obtained from a small window around the singular value transition region. In this regime, the active-space sizes are comparably small. This is promising since high accuracy for small active system sizes is one key requirement for successful embedding strategies.

### 3.3 ACE-of-SPADE—Automated, Consistent, and Even-Handed Active-Space Selection Scheme

The SPADE algorithm bridges the gap between accuracy and efficiency and provides a suitable framework to establish an even-handed approach. However, due to missing convergence with respect to the active-space size, the selection of appropriate consensus sets of MOs along the trajectory is still challenging. As shown above, the most reliable results are obtained by focusing on MO selections close to the singular value transition region. When dealing with metal clusters where a lot of MOs with similar singular values exist in the transition area, the unique partitioning based on the largest singular value gap becomes error-prone. On the one hand, the position of the largest gap along the reaction trajectory can change, which leads to unphysical discontinuities in the PES. On the other hand, even small deviations from the transition region reduce accuracy.

**Figure 4:**
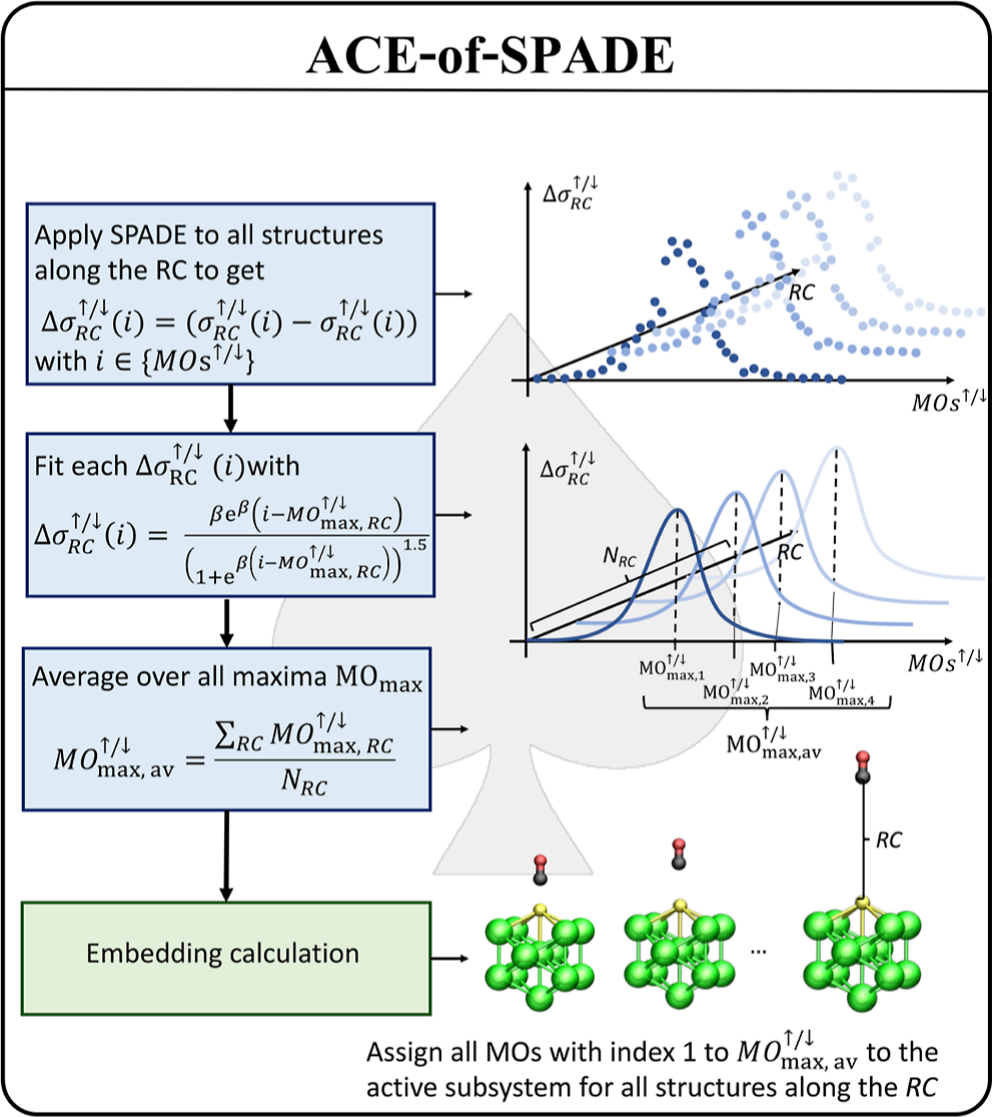
Flowchart of the ACE-of-SPADE active orbital space selection scheme for calculating the potential energy surfaces of chemical reactions. The images beside the flowchart illustrate the respective steps.

To solve these problems, we developed an automated, consistent, and even-handed active-space selection scheme based on the SPADE algorithm, called ACE-of-SPADE, for the successful application of PBET on metal nanoclusters and similarly challenging systems. This algorithm addresses the above-mentioned issues by performing iteratively three different steps (Figure 4): In the first step, the SPADE algorithm is applied to each individual structure along the reaction coordinate to get information about the prioritization of MOs based on the singular values. In the second step, the first derivative of the singular value distribution curve is fitted to the derivative of the square root of the Fermi function, , for each structure along the reaction coordinate separately. The variable *i* labels the MO index, and MO_max,RC_ describes the peak position of the fit function obtained from the singular value distribution at the reaction coordinate (RC). Note that this analysis is performed independently for the alpha and beta MOs, indicated by ↑/↓, respectively. In the case of metal systems, the singular values follow an almost continuous distribution in the transition area between 0 < σ_*i*_ < 1, due to the many near-degenerate MOs. This leads to a multipeak structure for the first derivative. The first derivative is fitted to a simple functional form with a single maximum to uniquely define where to partition the molecular space. Further, for reliable orbital selections for the entire reaction, information from all structures along the trajectory need to be considered. Due to the fitting procedure, the peak position depends not only on the position of individual singular values but also on the distribution of all singular values close to the transition area. Thus, the peak of the fit function indicates the most suitable system partition. All MOs with an index lower than that determined by the peak are assigned to be the active subsystem, while all the other MOs belong to the environment. The motivation for exploiting this functional form of the fit function comes from the analogy of interpreting the squared singular values as similar to occupation numbers.^55^ Although this approach is more stable for metallic systems than the automated active-space selection according to the largest gap of consecutive singular values, it cannot generally be excluded that the peak position changes distinctively along the trajectory. To compensate for this case, in a third step, the average values of all peak positions are calculated. This final average value indicates where the system is partitioned with respect to the requirements of each individual configuration along the whole trajectory. The last step ensures uniformity by taking the same set of MOs at each point of the reaction coordinate into account.

This method ensures even-handedness, as long as the hierarchy of the MOs defined by their singular values does not significantly change along the reaction coordinate. Although such a phenomenon has not been observed so far, it is still conceivable. This is problematic because a changing hierarchy can destroy the selection of a consensus set of the same MOs with respect to their increasing MO indexes. However, the (ACE-of-) SPADE provides a framework to address this issue. As shown in Figure 2, the evolution of the MOs can be traced and uniquely identified. If the singular values of MOs close to the transition area change dramatically along the RC, such that the hierarchy changes, then the corresponding MOs must be taken into account regardless of their final MO index. In this way, even-handedness is re-established. However, a detailed accuracy analysis must be carried out if such a phenomenon is to be observed in future studies.

Further, it is important to mention that the active space identified by the ACE-of-SPADE algorithm depends on the set of structures on which the selection was based. Choosing structures only around the minimum would result in an active space that is perfectly adapted to only this region. While this is not a problem of the ACE-of-SPADE algorithm itself, as it would still provide smooth curves for any selected range of structures, the accuracy when going beyond the predefined range might suffer. Therefore, we highlight the importance of the choice of this initial set of structures. This importance is exacerbated when the embedding method with the selected active space is to be used for structure optimizations since the calculated gradients might be faulty for structures that are too distinct from the initial set of structures. We recommend including structures for all significant points on the potential energy surface (equilibrium structures, transition states, and dissociation limits) and for regions where the energy changes strongly for small structural displacements. A detailed benchmark study on a variety of electronically different systems is currently being carried out in our laboratory to identify criteria for a meaningful selection of the initial set of structures.

### 3.4 Application and Verification: Convergence and Error Cancellation

**Figure 5:**
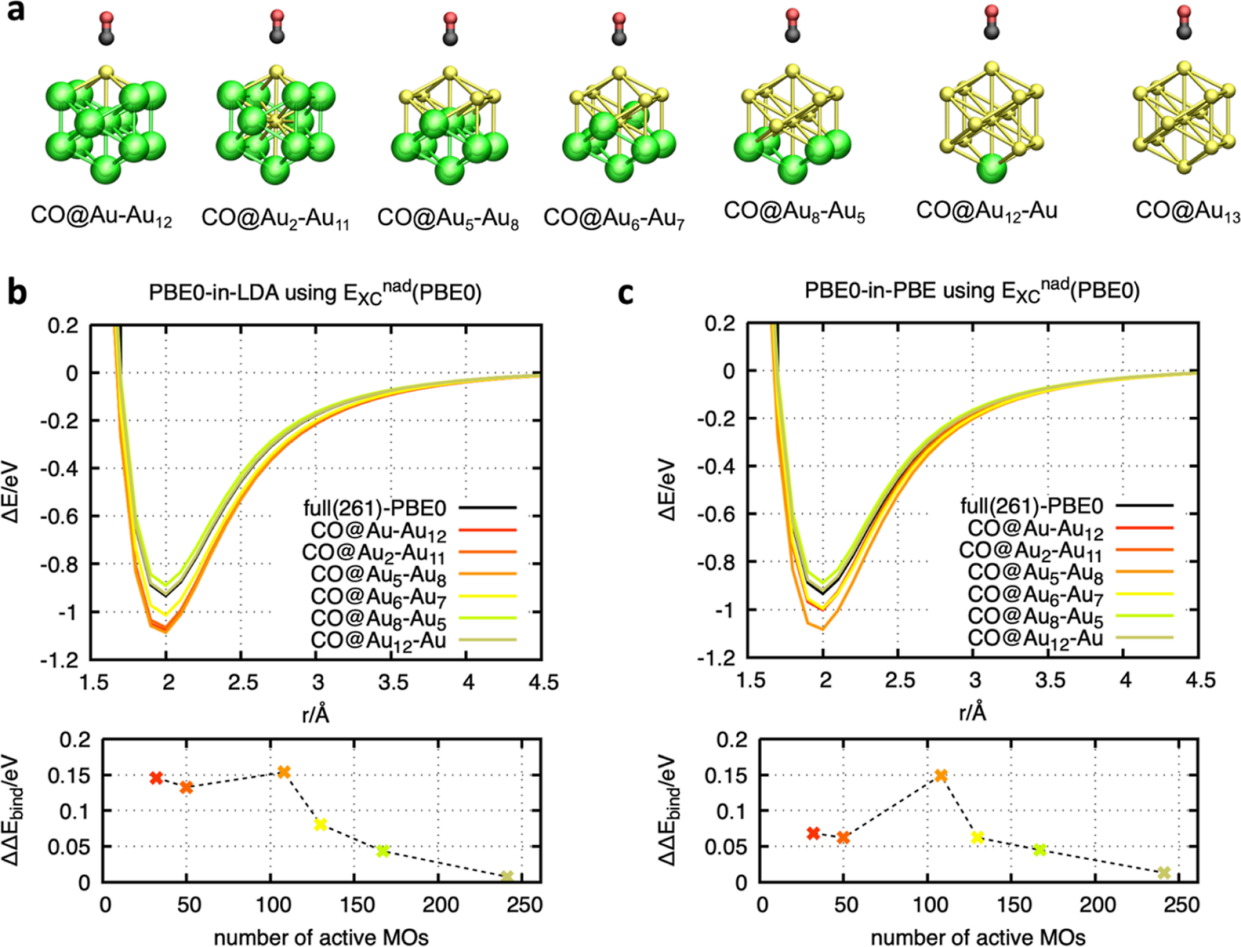
Analyzing the influence of the active cluster size on the binding energy. (a) Illustration of the different nanoclusters. Green-colored Au atoms represent the molecular environment, and the yellow Au atoms, black C atom, and red O atom describe the active core cluster. (b/c) Binding energy along the RC for the different clusters shown in (a). The top graphs show the dissociation curves, while the bottom plots show the error of the binding energy of the embedding method with respect to the full PBE0 reference (ΔΔ*E*_bind_ = Δ*E*_bind_^ACE-of-SPADE-PBET^ – Δ*E*_bind_^PBE0^). The plots in (b) are based on PBE0-in-LDA embedding, using PBE0 for the nonadditive exchange functional. The plots in (c) are based on PBE0-in-PBE embedding using PBE0 for the nonadditive exchange functional.

In order to demonstrate the reliability and validity of the ACE-of-SPADE active orbital space selection, the dissociation curve of CO@Au_13_ has been investigated by considering six different active atom selections (see Figure 5a). These clusters are calculated based on the two different functionals for the environmental systems, LDA (see Figure 5b) and PBE^80^ (see Figure 5c). The application of the ACE-of-SPADE selection scheme leads to smooth curves without any discontinuities for all configurations. Moreover, it should be emphasized that the active-space size is not sensitive to the method used for deriving the initial set of MOs (Figure 5 and Table S2). The analysis of the binding energy with respect to the reference curve at full PBE0 level shows only errors lower than 0.15 eV. Moreover, due to the consistent selection scheme, a convergence of accuracy with respect to the size of the active orbital space is generally achieved. Rather than due to fortuitous error cancellation, the convergence is caused by decreasing errors of the individual energetic contributions, i.e., from the active system, the environment, and the interaction terms.

**Figure 6:**
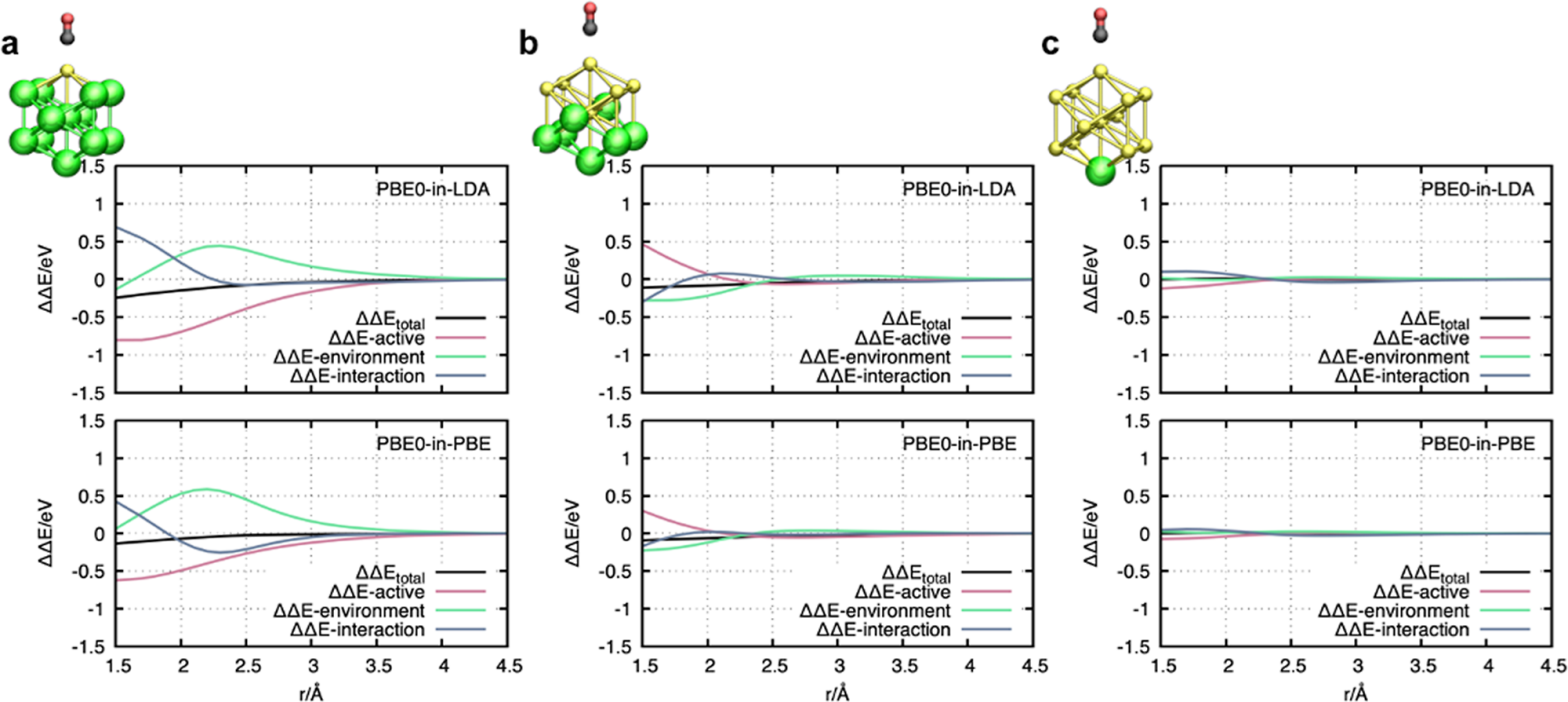
Individual error contributions for different systems with different active atom configurations. The black curves represent the total error given by the accumulation of individual contributions. The top diagrams show the calculated errors for treating the environment with the LDA functional, while the bottom diagrams show the environment with the PBE functional. (a) shows the errors for the cluster partitioning CO@Au–Au_12_, (b) CO@Au_6_–Au_7_ (c), and CO@Au_12_–Au.

The error of each contribution is calculated according to ΔΔ*E*_X_ (*r*) = Δ*E*_X_^ACE-of-SPADE-PBET^(*r*) – Δ*E*_X_^PBE0^ (*r*), where *X* labels the respective contribution: *X* ∈ {active, environment, and interaction}. The contributions for Δ*E*_X_^PBE0^ (*r*) are obtained from PBE0-in-PBE0 embedding calculations based on the same system partitioning as the ACE-of-SPADE embedding calculation. Figure 6 shows the errors of the different contributions for the three different active system sizes: CO@Au–Au_12_, CO@Au_6_–Au_7_, and CO@Au_12_–Au. The top image of each subfigure shows the error contributions and their total accumulation for considering the environment with the LDA functional, and the bottom image shows the errors for a calculation where the environment is treated with the PBE functional. This analysis shows that the surprisingly high accuracy, achieved with small cluster sizes, results from error compensations of large negative errors ΔΔ*E*_active_ (*r*), with smaller positive errors of both the environment ΔΔ*E*_environment_ (*r*) and the interaction contributions ΔΔ*E*_interactions_ (*r*) (see Figure 6a,b). The larger the active space, the smaller the individual error contributions (Figure 6c). Thus, increasing accuracy for increasing active systems is not coincidental but a result of decreasing individual errors as should be the case.

However, despite the observed convergence, the CO@Au_5_–Au_8_ cluster configuration constitutes an outlier in the dissociation curves shown in Figure 5. For this active cluster size, the individual error contributions are sufficiently large such that mutual error compensation is required for a (fortuitous) accurate result (see Figure S11). Apparently, due to a bad definition of active atoms, the error cancellation is poorly balanced, which leads to total errors of around 0.15 eV.

The observed convergence behavior, as well as the generally low errors and smooth and continuous curves, highlight the validity of the ACE-of-SPADE approach for the application of PBET on systems as challenging as metal nanoclusters. However, errors in small systems which originate from bad active atom definitions cannot be avoided completely, as discussed for the CO@Au_5_–Au_8_ partitioning.

### 3.5 Influence of the Non-additive Exchange Functional in PBET

**Figure 7:**
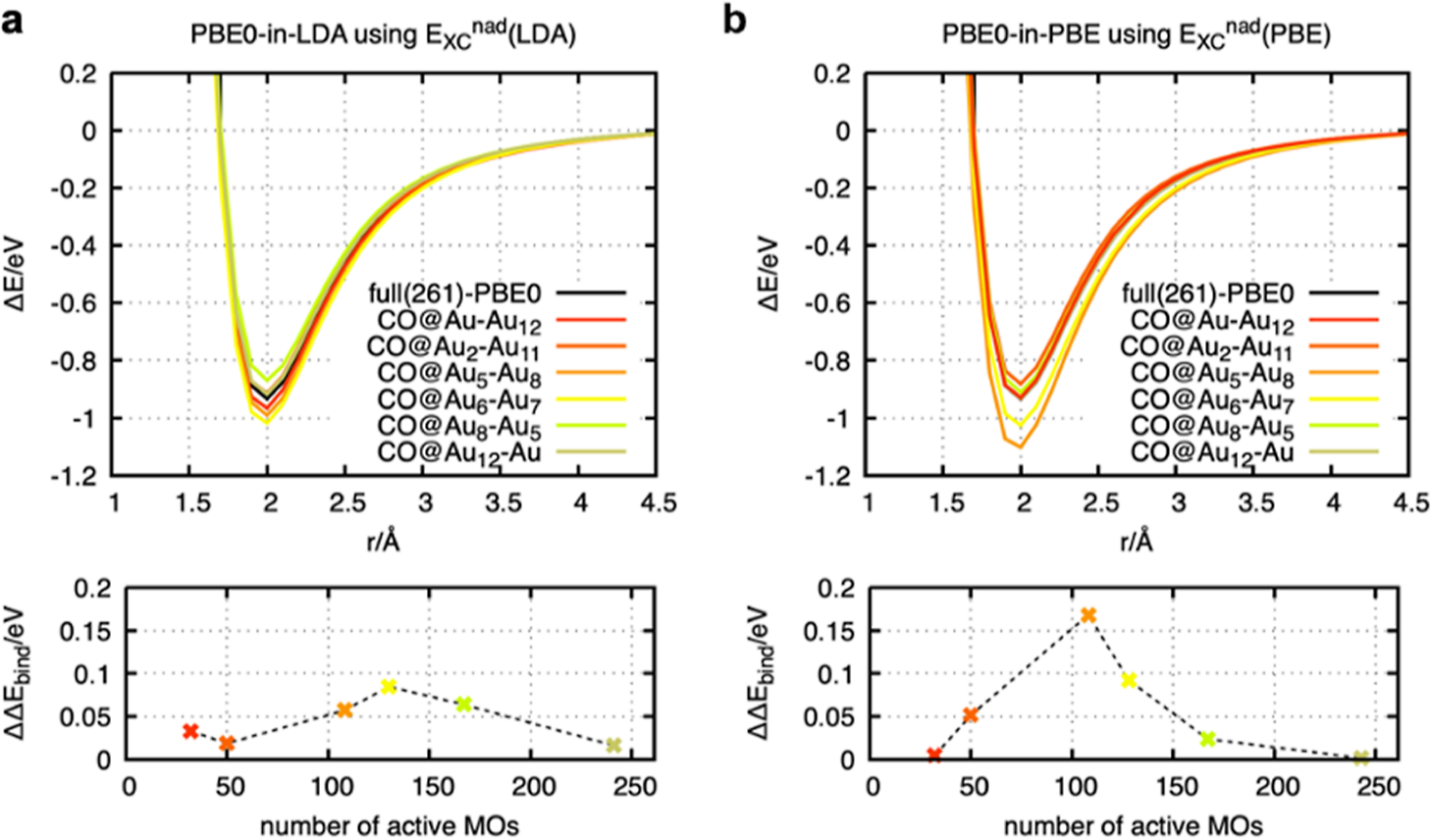
Top panels show the dissociation curves for different cluster configurations, and the bottom panels show the error of the binding energy of the embedding method with respect to the full PBE0 reference calculation (ΔΔ*E*_bind_ = Δ*E*_bind_^ACE-of-SPADE-PBET^ – Δ*E*_bind_^PBE0^). The plots in (a) are based on PBE0-in-LDA embedding, using LDA for the nonadditive exchange functional. The plots in (b) are based on PBE0-in-PBE embedding using PBE for the nonadditive exchange functional.

Analyzing results from calculations with different choices for the nonadditive exchange functional indicates that further improvements might be possible (see Figure 7). When the lower-ranked functional is used to describe the electron–electron exchange interactions between the active system and the environment, the accuracy for the smallest two active cluster configurations (CO@Au–Au_12_ and CO@Au_2_–Au_11_) increases dramatically. The errors are reduced to values lower than 0.05 eV (see Figure 7a) for using the LDA functional, while the PBE functional yields even errors tending toward zero for CO@Au–Au_12_ (see Figure 7b). Such reduced errors, particularly for the smallest clusters, are highly promising in the context of quantum embedding strategies because they indicate a high potential for dramatically reducing the numerical costs without loss of accuracy.

The analysis of the individual error contributions indicates that the reduced errors come from well-balanced error cancellation. The individual contributions are larger compared to the conventional PBET using the higher level of theory of the nonadditive exchange functional (see Figure S12). Although the error cancellation is well-balanced for the smallest systems, the errors for larger clusters such as CO@Au_5_–Au_8_ and CO@Au_6_–Au_7_ are slightly increased (see Figure 7). These systems are still in a regime where a low total error is observed due to error cancellation of the individual energy contributions. Since the error compensation is poor for these two clusters, the deviation from reference curves increases, and no convergence with respect to the cluster size is observed.

In summary, the dramatically reduced errors for small active subsystems in a metallic environment with the LDA functional for the nonadditive exchange contribution is a very promising observation. Further studies on a large selection of metallic clusters are currently being performed by our group.

## 4 Conclusions

In summary, we presented a systematic study of the application of projection-based embedding theory on metal nanoclusters. Our work highlights and analyzes inconsistencies and inaccuracies which arise from conventional active-space selection schemes. We have shown that active-space selection approaches, which are based on strict localizations and tend to converge to molecular orbitals with near-atomic character, cannot adequately partition the molecular system into active and environmental subsystems for a reaction coordinate. The SPADE algorithm is more robust since it preserves the delocalized character of the molecular orbitals of metal nanoclusters within the respective subsystem. However, we demonstrated that the selection criterion of the SPADE algorithm becomes problematic for metal nanoclusters due to the high number of (near-)degenerate molecular orbitals and that the automated selection according to the largest gap between consecutive singular values can lead to discontinuities in the energy profile along a reaction coordinate. Nevertheless, the application of the singular value decomposition procedure on the active subspace allows us to reliably rank the molecular orbitals and to follow the evolution of the molecular orbitals along a reaction coordinate. This can be exploited to define a consensus set of molecular orbitals as active orbital space, transforming the automated SPADE into an even-handed approach. The accuracy of the resulting potential energy surfaces depends strongly but nonmonotonically on the size of the active orbital space.

To address these issues, we developed an automated, consistent, and even-handed active-space selection approach based on the SPADE algorithm (ACE-of-SPADE) that allows the successful calculation of potential energy surfaces of metal nanoclusters by using embedding approaches. The ACE-of-SPADE algorithm considers the distribution of singular values at each point of the trajectory. In order to avoid inconsistencies from a large number of (near-)degenerate molecular orbitals, the system partitioning is conducted with respect to Fermi-type fit functions. Based on this partitioning, a set of molecular orbitals is selected which contributes on average most to the active system along the reaction coordinate and ensures both high accuracy and consistency.

We demonstrated the validity of this approach by applying it to a metal nanocluster with different definitions of active atoms. We showed that ACE-of-SPADE provides smooth and continuous energy reaction profiles. Further, our approach establishes a converging improvement in accuracy as the active orbital space sizes increase. The active-space partitioning according to the ACE-of-SPADE algorithm is a direct function of the active atoms selected. This observation is confirmed by a detailed analysis of the individual error contributions. With increasing active system sizes, individual errors decrease, which is an indicator of reliable embedding. However, for a poor selection of the active region, this convergence can be disturbed, particularly for small active systems. There, the individual error contributions are considerably large, but the fortuitous error cancellation is poor.

By contrast, we could also show that favorable error cancellation can result in high accuracy with low numerical effort. But in order to exploit the advantages of favorable error cancellation, further investigations on the impact of the selection of the active region are required. Even so, we stress that the errors for all calculations are relatively small, with values lower than 0.15 eV for the binding energy.

Since error compensation is crucial for accuracy in the case of small active regions, convergence must be checked manually for any application. However, because ACE-of-SPADE allows us to increase the accuracy by increasing the active region, users are given control over accuracy and efficiency. Our algorithm offers a reliable and systematically improvable embedding strategy, but balancing accuracy and computational demands remains the user’s responsibility. However, this is a general shortcoming of embedding schemes themselves, not an issue introduced by ACE-of-SPADE.

Our work introduces the ACE-of-SPADE approach as a reliable active-space selection approach for analyzing the potential energy surface of chemical reactions on metal nanoclusters and thus significantly contributes to the computational chemistry toolbox for heterogeneous catalysis.

## Supporting Information Available

Additional Information about the modification of the Serenity 1.4 code, detailed explanation about the implementation of ACE-of-SPADE, definition of the probe system, visualization of MOs localized using conventional localization methods, additional information about the sensitivity of the active-space partitioning, singular value distribution of the CO@Au–Au_12_ cluster, and supplementary error compensation plots to Figures 5 and 7 (PDF)
