# Supplementary material for: Automated, Consistent, and Even-Handed Selection of Active Orbital Spaces for Quantum Embedding
Source: J Chem Theory Comput. 2023 Sep 29;19(19):6643–55. doi: 10.1021/acs.jctc.3c00653 (PMC10569175; doi:10.1021/acs.jctc.3c00653)
Supplement: Supplementary file 1 — ct3c00653_si_001.pdf [file ct3c00653_si_001.pdf]

Supporting Information

Automated, Consistent, and Even-handed Selection of Active Orbital Spaces for Quantum Embedding

Elena Kolodzeiski and *Christopher J. Stein*

Technical University of Munich, TUM School of Natural Sciences, Department of Chemistry, Lichtenbergstr. 4, D-85748 Garching, Germany

Contents

1. **Serenity Code Modifications**
2. **Definition of the Test System**
3. **Conventional Orbital Localizations**
4. **Impact of the Initial Orbital Choice and Structural Derivations**
5. **Singular Value Distributions**
6. **Error Compensation**

1. Serenity Code Modifications

In order to manually select where to partition the active and environmental system, modifications on the SPADE algorithm as implemented in the open-source code Serenity 1.4 (https://github.com/qcserenity/serenity/releases/tag/1.4.0) were required. Therefore, the file SPADEAlgorithm.cpp in the directory SERENITY_DIR/src/analysis/orbitalLocalization/SPADEAlgorithm.cpp has been changed. The main modification is given by reading and writing the “LastActiveOrbital_up.csv” and “LastActiveOrbital_down.csv” files. These files contain the integer number of actively treated orbitals for the alpha and beta MOs, respectively. Another small adaption that should be mentioned is made by writing out the singular value distribution. The output files are named “sigma_orb_up.txt” and “sigma_orb_down.txt”. The modified code is given below in Figure S1. The modified parts are highlighted in green.

The above-mentioned files are required for conducting the ACE-of-SPADE algorithm. In the following, our implementation of the ACE-of-SPADE approach will be presented. The calculation can be split into three parts. In the first step, for all structures along the reaction pathway, the SPADE algorithm is applied with the help of the Serenity program. In Figure S2 an exemplary serenity input is shown. With this input file, the Serenity program conducts the SPADE analysis for one given configuration and writes out the singular value distribution for the alpha and beta MOs (“sigma_orb_up.txt” and “sigma_orb_down.txt”), due to our modification. In the second step, post-processing takes place, extracting information about the preferred system partitioning from the “sigma_orb_up.txt” and “sigma_orb_down.txt” files. To do so, all obtained singular value distributions are fitted with the first derivative of the square root of the Fermi-type function (see Figure 3). This is carried out with the python script “fit_curve.py” (see Figure S3, tested using python 3.11). This script writes out the peak position of the different structures into individual “lastActiveOrbital_up.csv” and

“lastActiveOrbital_down.csv” files. Subsequently, these files are read by the script “calc_av_of_peaks.py” (see Figure S4). This script reads out all the peak positions, calculates the average value and writes it to new “lastActiveOrbital_up.csv” and “lastActiveOrbital_down.csv” files. When stored in the directory, where the serenity calculation will be performed, this file allows the manipulation of the system partitioning with respect to the integer numbers stored in the *.csv files in a subsequent serenity embedding calculation. The latter is the last step. Based on the “lastActiveOrbital_up/down.csv” files, the actual embedding calculation can be performed for all structures along the reaction coordinate. One representative serenity input file, required for this, is shown in Figure S5.

```
#include "analysis/orbitalLocalization/SPADEAlgorithm.h"
#include "basis/BasisFunctionMapper.h"            //Block selection in coefficient matrix.
#include "data/OrbitalController.h"               //Coefficients and orbital update.
#include "data/matrices/CoefficientMatrix.h"      //Coefficient matrix.
#include "data/matrices/MatrixInBasis.h"          //Overlap matrix.
#include "integrals/OneElectronIntegralController.h" //Overlap integrals for symmetric orthogonalization.
#include "io/FormattedOutputStream.h"             //Filtered output streams.
#include "math/linearAlgebra/MatrixFunctions.h"   //Matrix sqrt
#include "misc/SerenityError.h"                   //Error messages
#include "system/SystemController.h"              //System controller defintion.
#include <fstream>
namespace Serenity {
template<Options::SCF_MODES SCFMode>
SPADEAlgorithm<SCFMode>::SPADEAlgorithm(std::shared_ptr<SystemController> supersystem,
std::shared_ptr<SystemController> activeSystem): _supersystem(supersystem), _activeSystem(activeSystem) {}

template<Options::SCF_MODES SCFMode> SpinPolarizedData<SCFMode, Eigen::VectorXi> SPADEAlgorithm<SCFMode>::run()
{
 auto orbitalController = _supersystem->getActiveOrbitalController<SCFMode>();
 const MatrixInBasis<RESTRICTED>& S = _supersystem->getOneElectronIntegralController()->getOverlapIntegrals();
 const Eigen::MatrixXd sqrtS = mSqrt_Sym(S);
 CoefficientMatrix<SCFMode> coefficients = orbitalController->getCoefficients();
 auto nOcc = _supersystem->getNOccupiedOrbitals<SCFMode>();
 SpinPolarizedData<SCFMode, Eigen::VectorXi> assignment;
 BasisFunctionMapper basisFunctionMapper(_supersystem->getBasisController());
 auto projection = basisFunctionMapper.getSparseProjection(_activeSystem->getBasisController());
 std::ofstream outfile1("sigma_orb_up.txt");
 std::ofstream outfile2("sigma_orb_down.txt");
 int spinstate=0;
 for_spin(coefficients, nOcc, assignment) {
  const Eigen::MatrixXd oldC = coefficients_spin.leftCols(nOcc_spin).eval();
  const Eigen::MatrixXd d = oldC * oldC.transpose();
  const Eigen::MatrixXd orthoC = sqrtS * oldC;
  const Eigen::MatrixXd orthoC_A = *projection * orthoC;
  // Calculation of the full matrix V is necessary for the orbital rotation.
  Eigen::JacobiSVD<Eigen::MatrixXd> svd(orthoC_A, Eigen::ComputeFullU | Eigen::ComputeFullV);
  const Eigen::MatrixXd rightSingularVectors = svd.matrixV();
  const Eigen::VectorXd singularValues = svd.singularValues();
  const Eigen::MatrixXd newC = (oldC * rightSingularVectors).eval();
  const Eigen::MatrixXd d2 = newC * newC.transpose();
  if ((d - d2).array().abs().sum() > 1e-10)
   throw SerenityError("Density matrix changed during SPADE orbital construction.");
  coefficients_spin.leftCols(nOcc_spin) = newC;
  unsigned int lastActiveOrbital = nOcc_spin;
  double largestDifference = 0.0;
  for (unsigned int iOrb = 1; iOrb < singularValues.size(); iOrb++) {
   double singularValueDifference = std::fabs(singularValues[iOrb - 1]  - singularValues[iOrb]);
   if (spinstate==0){ outfile1 << iOrb << "   " << singularValues[iOrb] << "   " << singularValueDifference  << " \n";}
   if (spinstate==1){ outfile2 << iOrb << "   " << singularValues[iOrb] << "   " << singularValueDifference << " \n";}
   if (singularValueDifference > largestDifference) {
    largestDifference = singularValueDifference;
    lastActiveOrbital = iOrb;
   }
  } // for iOrb
  assignment_spin = Eigen::VectorXi::Constant(nOcc_spin, 1);
  if (spinstate==0){
        std::ifstream ifile2("lastActiveOrbital_up.csv");
        if (ifile2){ ifile2 >> lastActiveOrbital;}
        ifile2.close();
  }
  if (spinstate==1){
        std::ifstream ifile2("lastActiveOrbital_down.csv");
        if (ifile2){ ifile2 >> lastActiveOrbital;}
        ifile2.close();
  }
  assignment_spin.head(lastActiveOrbital) = Eigen::VectorXi::Zero(lastActiveOrbital);
  spinstate++;
 };
 // TODO: Allow the splitting of core and non-core orbitals.
 orbitalController->updateOrbitals(coefficients, _supersystem->getActiveOrbitalController<SCFMode>()->getEigenvalues());
 outfile1.close();
 outfile2.close();
 return assignment;
}
template class SPADEAlgorithm<Options::SCF_MODES::RESTRICTED>;
template class SPADEAlgorithm<Options::SCF_MODES::UNRESTRICTED>;
} /* namespace Serenity */
```

Figure S1. Modified code of the Serenity 1.4 package SPADEAlgorithm.cpp. The modified parts are highlighted in green.

```
+system
    name Au13_CO
    geometry Au13_CO.xyz
    method dft
    +dft
        functional LDA
    -dft
    +basis
        label DEF2-SVP
    -basis
    charge 0
    spin 1
-system

+system
    name Au_CO
    geometry Au_CO.xyz
    method dft
    +dft
      functional PBE0
    -dft
    +basis
      label DEF2-SVP
    -basis
    charge 0
    spin 1
-system                                   page 1
```

```
+system
    name Au12
    geometry Au12.xyz
    method dft
    +dft
        functional LDA
    -dft
    +basis
      label DEF2-SVP
    -basis
    charge 0
    spin 0
-system

+task SCF
    system Au13_CO
-task

+task SPLIT
    act Au13_CO
    env Au_CO
    env Au12
    systemPartitioning SPADE
    printLevel DEBUGGING
-task
                                          page 2
```

Figure S2. Exemplary Serenity input file for conducting the SPADE with the modified SPADEAlgorithm.cpp code. Starting Serenity with this input writes out the singular value distribution to the files "sigma_orb_up.txt" and "sigma_orb_down.txt".

```
import numpy as np
from scipy.optimize import curve_fit

def deriv_fermi(x, x0, beta, scale):
    numinator=scale*beta*np.exp(beta*(x-x0))
    denominator=(1.+np.exp(beta*(x-x0)))**1.5
    return numinator/denominator


opt_id=[]
for spin in ["up", "down"]:
    matrix=[]
    infile=open("sigma_orb_"+spin+".txt", "r")
    id_max=0
    value=0
    i=0


    x_data=[]
    y_data=[]
    for line in infile:
        x_data.append(float(line.strip().split()[0]))
        y_data.append(float(line.strip().split()[2]))
        if float(line.strip().split()[2]) > value:
            value=float(line.strip().split()[2])
            id_max=i
        i+=1
    infile.close()


    popt, pcov = curve_fit(deriv_fermi, x_data, y_data, p0=[id_max, 1., 1.])
    opt_id.append(float(popt[0]))

for spin in ["up", "down"]:
    outfile=open("lastActiveOrbital_"+spin+".csv", "w")
    if spin == "up": outfile.write(str(opt_id[0]+1)) #+1 because the modified serenity code just reads
the number of active MO, not the last index, which starts with index 0.
    if spin == "down": outfile.write(str(opt_id[1]+1)) #see explanation above
    outfile.close()
```

Figure S3. The “curve_fit.py” script. This script reads the singular value distributions stored in “sigma_orb_up/down.txt” and fits this curve with the first derivative of the square root of the Fermi-type function. The peak position is then saved as float in the files “lastActiveOrbital_up/down.csv”. By placing these output files in the directory where the serenity calculation is conducted, the system partitioning is manipulated with respect to the information defined in these files.

```python
import numpy as np
import os


tmp_input=input("start values: ")
start_val=int(tmp_input.strip().split()[0])
end_val=int(tmp_input.strip().split()[1])


lastActiveOrbitals=0
for spin in ["up","down"]:
  act_orb=[]
  lastActiveOrbital=0
  for i in range(start_val, end_val):
    filename="./"+str(i)+"/lastActiveOrbital_"+spin+".csv"
    if os.path.isfile(filename):
        infile=open(filename,"r")
        for line in infile:
            tmp=float(line.strip().split()[0])
            act_orb.append(tmp)
        infile.close()
        If tmp > lastActiveOrbital: lastActiveOrbital=tmp
        else: pass
    if lastActiveOrbital==0:
        print("THERE IS NOT A SINGLE LASTACTIVEORBITAL FILE")
        exit()
lastActiveOrbital=np.average(act_orb)
outfile = open("lastActiveOrbital_"+spin+".csv","w")
outfile.write(str(int(lastActiveOrbital)))
outfile.close()
```

Figure S4. The “calc_ac_of_peaks.py” script. This script requires that the files “lastActiveOrbital_up.csv” and “lastActiveOrbital_down.csv” are saved in different directories numbered with respect to the corresponding structure along the trajectory. The script reads iteratively all “lastActiveOrbital_up/down.txt” files and calculates the average value. The integer number of the average value is then written to new “lastActiveOrbital_up/down.txt” files. This is the basis for manipulating the system partitioning during a subsequent embedding calculation using the modified version of Serenity.

```
+system
    name Au13_CO
    geometry Au13_CO.xyz
    method dft
    +dft
        functional LDA
    -dft
    +basis
        label DEF2-SVP
    -basis
    charge 0
    spin 1
-system

+system
    name Au_CO
    geometry Au_CO.xyz
    method dft
    +dft
        functional PBE0
    -dft
    +basis
        label DEF2-SVP
    -basis
    charge 0
    spin 1
-system

+system
    name Au12
    geometry Au12.xyz
    method dft
    +dft
        functional LDA
    -dft
    +basis
        label DEF2-SVP
    -basis
    charge 0
    spin 0
-system                                page 1
```

```
+task SCF
    system Au13_CO #optional: the previous
                   calculated orbitals can be
                   read to skip this calculation
                   and to accelerate the whole
                   procedure.
-task

+task SPLIT
    act Au13_CO
    env Au_CO
    env Au12
    systemPartitioning SPADE
    printLevel DEBUGGING
-task

+task FDE
    act Au_CO
    env Au12
    +EMB
        naddXCFunc PBE0
        embeddingMode FERMI
    -EMB
    calculateEnvironmentEnergy True
    printLevel DEBUGGING
-task
                                       page 2
```

Figure S5. Exemplary Serenity input file for conducting the final embedding calculation with the modified SPADEAlgorithm.cpp code. Starting Serenity with this input and the previously “lastActiveOrbital_up/down.csv”-files stored in the directories where Serenity is executed, reads in where the system partitioning occurs and conducts the separation of active and environmental systems accordingly.

2. Definition of the Test System

Figure S6 shows the CO@Au_{13} test system used in this study. The minimum structure used as a template for all calculations is given below in Table S1.

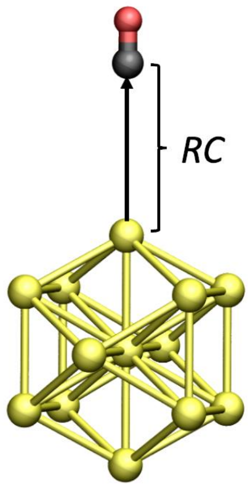

Figure S6. Illustration of the test system CO@Au_{13}. The reaction coordinate *RC* is defined by the vector pointing from the top Au atom to the C atom of the substrate.

15			
C	3.5456393166760583	0.6083152873033600	4.7395663983811070
O	3.5456393166760583	0.6083152873033600	5.8748736779578070
Au	3.5456393166760583	0.6083152873033600	2.7395663983811067
Au	3.5456393166760583	0.6083152873033600	7.474353716937067e-07
Au	1.0812310140118795	1.0746189400920991	1.4128783326884087
Au	3.0793180446227852	3.0728191605219006	1.4129248011236335
Au	4.0119418581793770	-1.856085882304913	1.4128740516959597
Au	6.0101354931901465	0.1419948119741670	1.4129207605189390
Au	1.6085409127884487	-1.328784654606081	5.673546180810722e-06
Au	5.4828962177637140	2.5455709125561348	-2.72215299647758e-06
Au	1.0812135215815302	1.0746195529897853	-1.4128812237879000
Au	4.0119424038699960	-1.856103422579450	-1.4128741879203162
Au	3.0793120535051948	3.0728310848040390	-1.4129375215170563
Au	6.0101468807459850	0.1419884309802613	-1.4129303290171256
Au	3.5456334264436937	0.6083087693573925	-2.7395995321356534

Table S1. Coordinates of the probe system CO@Au_{13} at 2.0 Å used as a template for all calculations.

3. Conventional Orbital Localizations

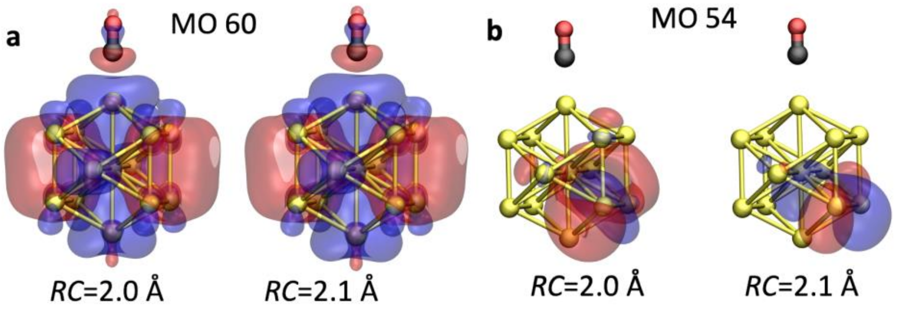

Figure S7. MOs after orbital localization using IBO. a) highlights that the atom-orbital-like characteristics are not always achieved. The image shows the MO with index 60 for a reaction coordinate *RC*=2.0 Å and *RC*=2.1 Å. b) shows that the localization might converge to different electronic minima for slightly different structures. Shown are the MO with index 54. Note, orbitals with different indices have also been compared here for better agreement, but none were found. The isosurfaces highlighted in blue and red correspond to isovalues of ± 0.0025, respectively.

4. Impact of the Initial Orbital Choice and Structural Derivations

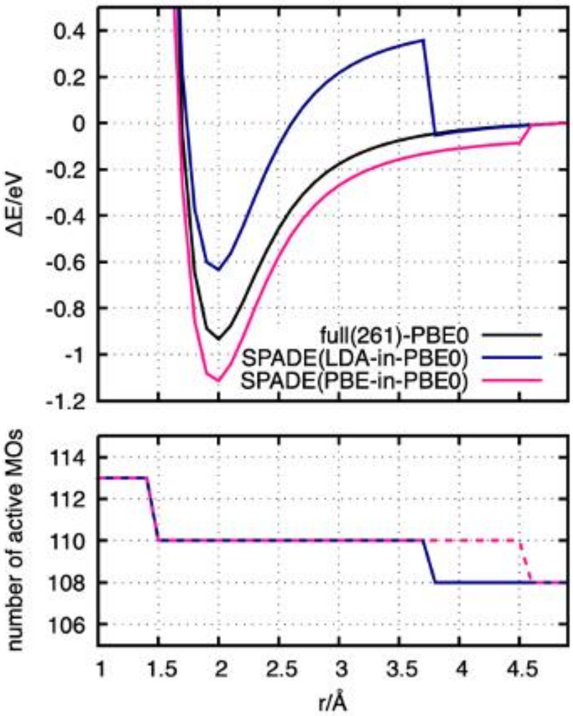

Figure S8. Impact of the exchange functional on the active space selection using the SPADE algorithm. The top image shows the calculated dissociation curve using the SPADE algorithm. The bottom plot shows the size of the active orbital space. Both functionals suffer from discontinuities in the dissociation curve, which are related to a changing active space size.

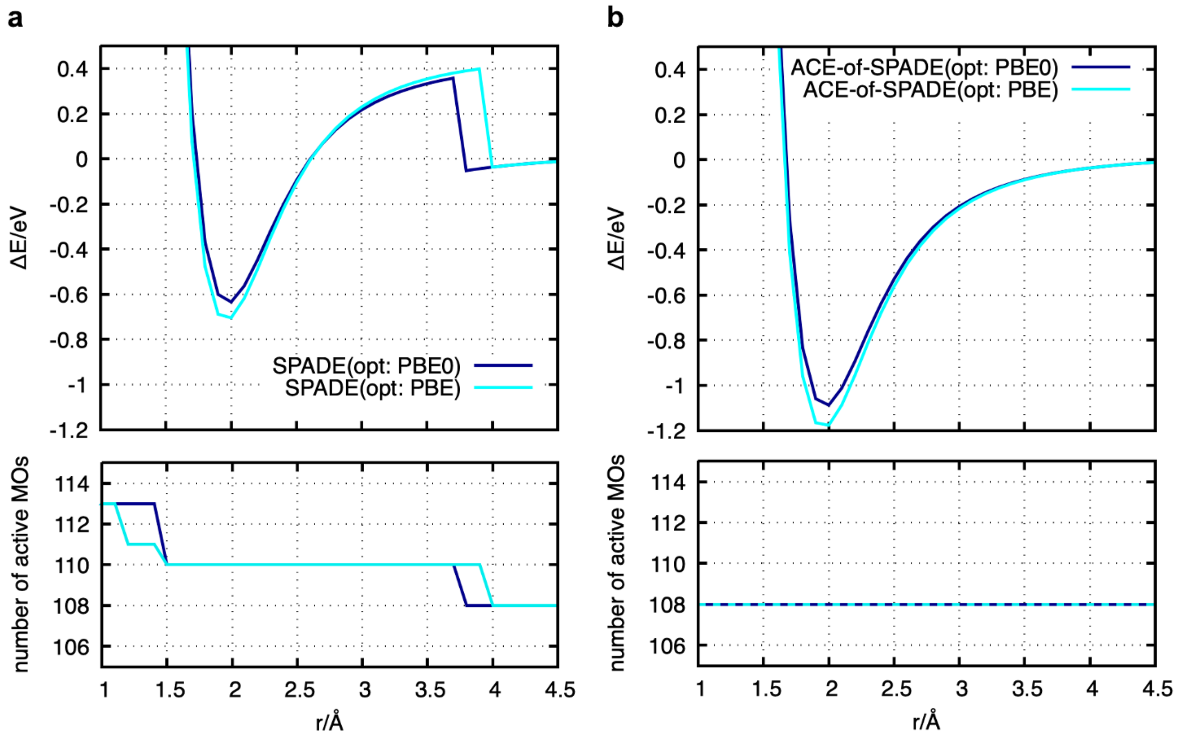

Figure S9. Impact of the method for the structural optimization of metal nanocluster on the active-space selection. The dark-blue curves belong to the PBE0 preoptimized structures used in the main manuscript and the cyan curves belong to the PBE preoptimized structures. a) The left top image shows the dissociation curve and the number of MOs selected as active by the original SPADE algorithm. The discontinuity discussed in the main manuscript occurs for slightly different configurations, due to a change in the active orbital space size. b) The right top image shows the dissociation curve after applying the ACE-of-SPADE algorithm. The active space size is constant, which leads to continuous curves. The slightly different geometries have no impact on the qualitative behavior of the electronic-structure properties and the selected orbitals.

5. Singular Value Distribution

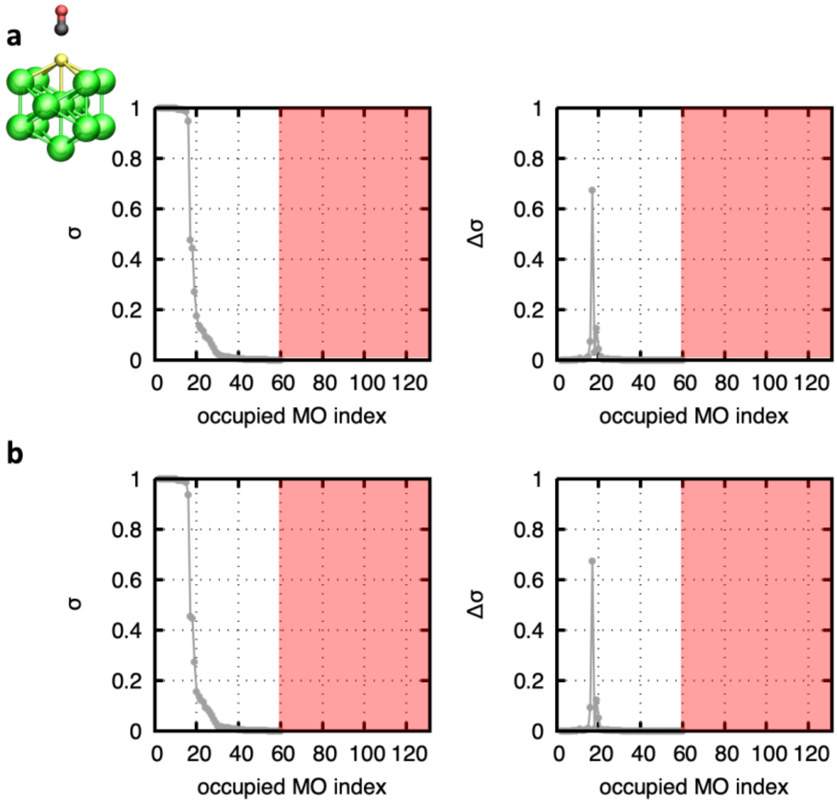

Figure S10. Plots of the singular value σ_i distribution (left panel) and the difference of consecutive MOs with respect to the MO index ($\Delta\sigma_i = \sigma_i - \sigma_{i-1}$) for the CO@Au-in-$Au_{12}$ nanocluster (right panel). a) shows the distribution of the alpha and b) of the beta MOs. The red areas are not subject to the singular value decomposition procedure.

	LDA	PBE
CO@Au-Au_{12}	32	32
CO@Au_2-Au_{11}	50	50
CO@Au_5-Au_8	108	108
CO@Au_6-Au_7	128	128
CO@Au_8-Au_5	176	176
CO@Au_{12}-Au	243	243
CO@Au_{13}	261	261

Table S2. Active space size for different system partitions using different exchange functionals. The number of active MOs are shown.

6. Error Compensation

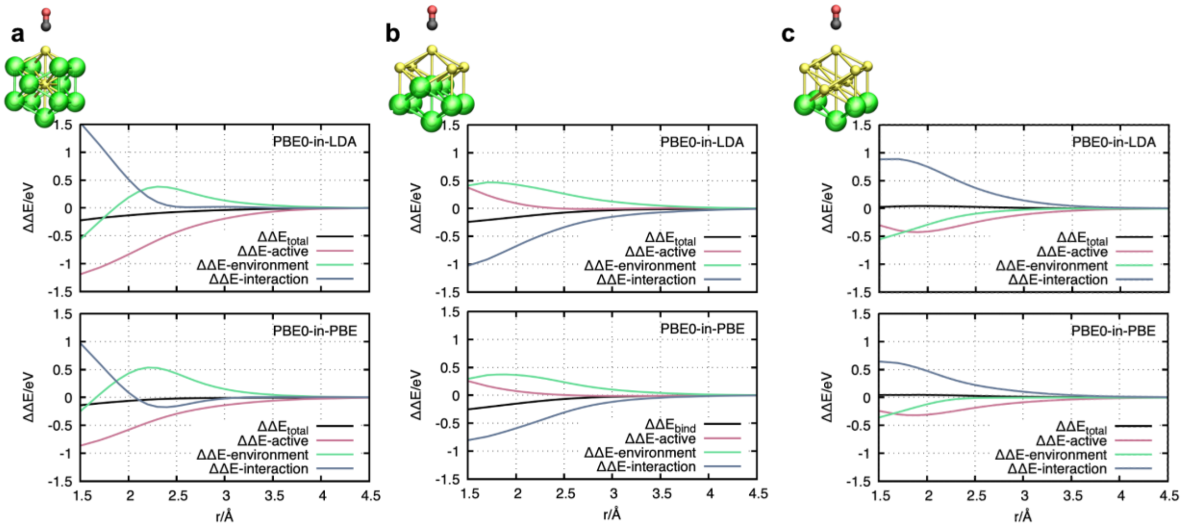

Figure S11. Individual error contributions for different systems with different active atom configurations. The black curves represent the total error given by the accumulation of individual contributions. The top diagrams show the calculated errors for treating the environment with the LDA functional, while the bottom diagrams show the environment with the PBE functional. The non-additive exchange interactions are described using PBE0. a) shows the errors for the cluster partitioning $CO@Au_2$-Au_{11}, b) for $CO@Au_5$-Au_8 and c) for $CO@Au_8$-Au_5.

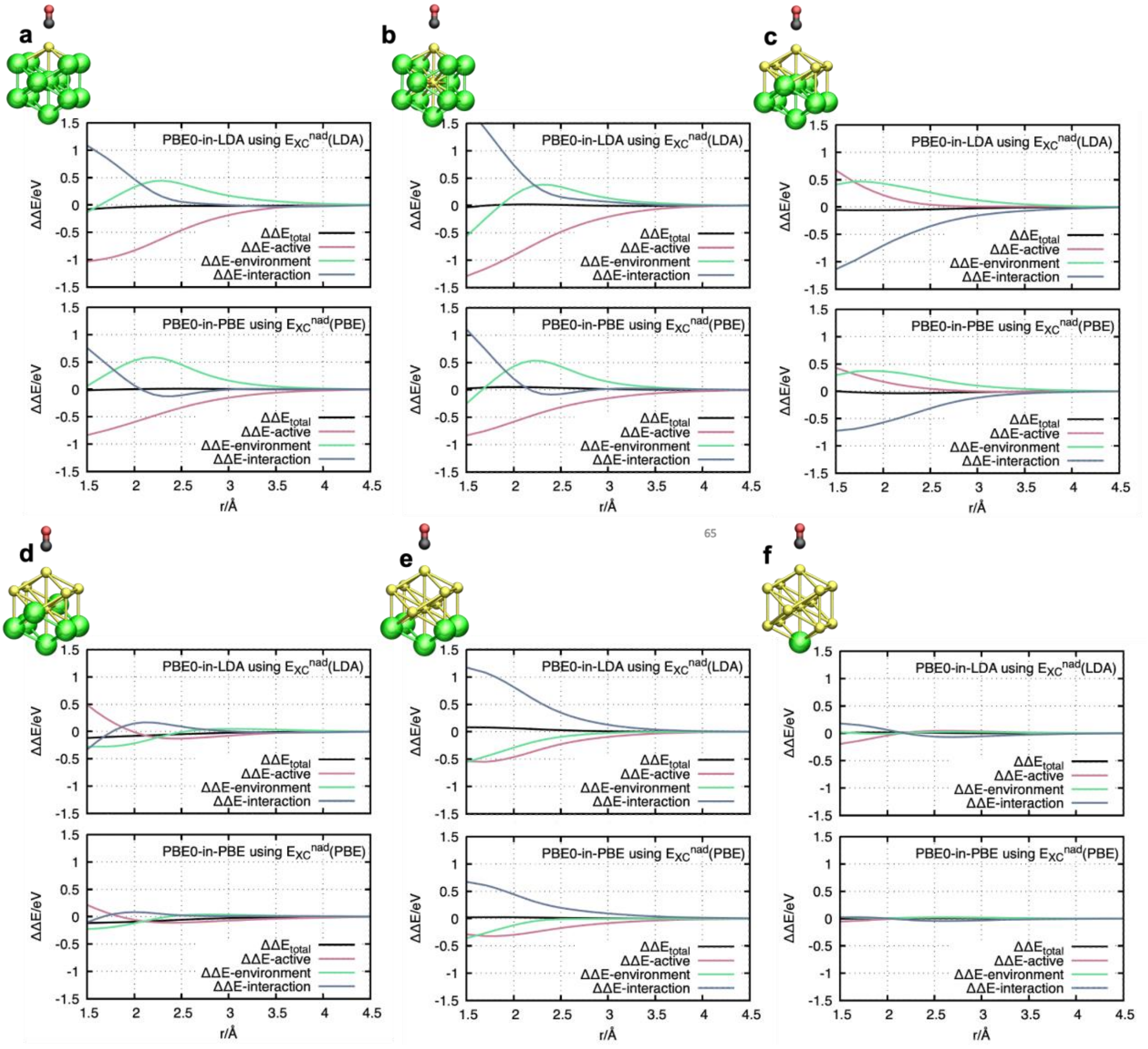

Figure S12. Individual error contributions for different systems with different active atom configurations. The black curves represent the total error given by the accumulation of individual contributions. The top diagrams show the calculated errors for treating the environment and the non-additive exchange interaction with the LDA functional, while the bottom diagrams show the environment and non-additive exchange interactions with the PBE functional. a) shows the errors for the cluster partitioning CO@Au-Au_{12}, b) for CO@Au_2-Au_{11}, c) for CO@Au_5-Au_8, d) for CO@Au_6-Au_7, e) for CO@Au_8-Au_5 and f) for CO@Au_{12}-Au
